# Signatures of reproductive events on blood counts and biomarkers of inflammation: Implications for chronic disease risk

**DOI:** 10.1371/journal.pone.0172530

**Published:** 2017-02-24

**Authors:** Daniel W. Cramer, Allison F. Vitonis

**Affiliations:** 1 Obstetrics and Gynecology Epidemiology Center, Department of Obstetrics and Gynecology, Brigham and Women’s Hospital, Boston, Massachusetts, United States of America; 2 Harvard Medical School, Boston, Massachusetts, United States of America; 3 Department of Epidemiology, Harvard School of Public Health, Boston, Massachusetts, United States of America; Vanderbilt University Medical Center, UNITED STATES

## Abstract

Whether inflammation mediates how reproductive events affect chronic-disease risk is unclear. We studied inflammatory biomarkers in the context of reproductive events using National Health and Nutrition Examination Survey (NHANES) data. From 15,986 eligible women from the 1999–2011 data cycles, we accessed information on reproductive events, blood counts, C-reactive protein (CRP), and total homocysteine (tHCY). We calculated blood-count ratios including: platelet-lymphocyte (PLR), lymphocyte-monocyte (LMR), platelet-monocyte (PMR), and neutrophil-monocyte (NMR). Using sampling weights per NHANES guidelines, means for counts, ratios, or biomarkers by reproductive events were compared using linear regression. We performed trend tests and calculated p-values with partial sum of squares F-tests. Higher PLR and lower LMR were associated with nulliparity. In postmenopausal women, lower PMR was associated with early age at first birth and higher NMR with later age at and shorter interval since last birth. Lower PNR and higher neutrophils and tHCY were associated with early natural menopause. In all women, the neutrophil count correlated positively with CRP; but, in premenopausal women, correlated inversely with tHCY. Reproductive events leave residual signatures on blood counts and inflammatory biomarkers that could underlie their links to chronic disease risk.

## Introduction

Recognition of the role of inflammation in the pathogenesis of chronic disease has led to interest in biomarkers of inflammation. Elevated white blood cell count (WBC) is a classic biomarker of inflammation. It is associated with increased risk of death from all causes, cardiovascular disease (CVD), and cancer, even after adjustment for confounders such as smoking [1–3]. In a cohort of elderly women, elevated WBC and neutrophils and lower lymphocytes at baseline were associated with an increased risk of death over the next 5 years [4]. This suggests the neutrophil-to-lymphocyte ratio (NLR) may also be a good biomarker of inflammation; and, indeed, an elevated NLR correlates with severity and prognosis for CVD, cancer, and other diseases [5, 6]. There has also been interest in the lymphocyte-to-monocyte ratio (LMR) and the platelet-to-lymphocyte ratio (PLR) as inflammatory biomarkers [7, 8]. Besides predicting disease risk or severity, differential counts and ratios derived from them also correlate with demographic and lifestyle factors such as age, sex, race, body mass index (BMI), smoking, and diet [9–11], suggesting inflammatory biomarkers do not simply signal end-stage illness but may also be involved in disease pathogenesis.

Reproductive events, including ages at menarche and menopause and number and timing of births also affect chronic disease risk. It is widely assumed that estrogen excess or deficiency explains this, especially for diseases where occurrence changes after menopause or risk in women predominates, such as CVD or autoimmune disorders. However, at least for the various autoimmune disorders, occurrence by age does not consistently track with menarche and menopause and responses to pregnancy and exogenous estrogen also vary [12]. This suggests that inflammatory/immune pathways may also be involved in associations between reproductive events and chronic disease. Surprisingly, the effects of past reproductive events on blood counts and inflammatory markers have been an infrequent topic of research.

The National Health and Nutrition Examination Survey (NHANES) is an important source of information on blood counts, inflammatory biomarkers derived from blood counts, and reproductive events. In subsets of NHANES participants, data on the serum inflammatory biomarkers, C-reactive protein (CRP), and total homocysteine (tHCY) are also available. Papers from NHANES indicate total WBC, neutrophils, and lymphocytes decrease with age while NLR, CRP, and tHCY increase [10, 13–15]. Compared to non-Hispanic whites and Hispanics, non-Hispanic blacks have lower total WBCs and neutrophils [13]. Greater BMI and current smoking lead to higher counts and inflammatory biomarkers [13]. In this study, we focus on how reproductive events affect blood counts and inflammatory biomarkers derived from blood counts using data from women who participated in NHANES. Serum CRP and tHCY are included to determine how they are affected by reproductive events and their correlation with blood counts.

## Methods

The NHANES Division, within the U.S. Centers for Disease Control and Prevention, began in 1999 with 2-year data collection cycles. In each cycle, about 5,000 people are selected and enrolled using a complex, multistage probability sampling design to create a representative sample of the civilian, non-institutionalized US population. Participants provide health-related data via questionnaires, physical exams, and laboratory assessments. People age 60 years and older, African Americans, and Hispanics are over-sampled. Health interviews are conducted in participants’ homes and exams in mobile examination centers where biologic samples are also collected. In all cycles, an automated determination of complete blood count (CBC) was performed using the Coulter method with different-generation Coulter Counters; and C-reactive protein (CRP) was measured in six cycles using Latex enhanced nephelometry on the Behring Nephelometer II Analyzer [16]. Total plasma homocysteine (tHCY) was measured in four survey cycles using high-performance liquid chromatography or commercial fluorescence polarization immunoassay on different-generation Abbott systems [17].

Use of de-identified data, available to the public, was deemed Not Human Research by Partners Human Research Committee, IRB Human Subjects Board; 2015P000935. We accessed publically available data from 1999 through the 2011 cycles on women 20 years of age or older. We excluded women who had incomplete CBC data (n = 2239) and those who did not complete the reproductive-health questionnaire or had unknown menopausal status (n = 1533), for a final sample of 15,986 women. From the CBC data, we accessed total WBC, lymphocytes, neutrophils, monocytes, hemoglobin, and platelets. Basophils and eosinophils were not included because of insufficient variability in the distributions. Women were classified as premenopausal if they reported regular periods in the past year or normally irregular cycles. Women were classified as postmenopausal if they reported no periods in the past year due to menopause or had a bilateral salpingo-oophorectomy (BSO) alone or at the time of hysterectomy. Women who reported no periods in the past year because of a hysterectomy (without a BSO) or because of a medical condition or treatment were classified as premenopausal if, at survey, they were age <50 and postmenopausal if age ≥50. Age at natural menopause was assigned as the age at last period for those who either never had a BSO or had a BSO after their periods stopped.

### Statistical analysis

We calculated the following ratios: platelets-neutrophils (PNR), platelets-lymphocytes (PLR), platelets-monocytes (PMR), neutrophils-lymphocytes (NLR), neutrophils-monocytes (NMR), and lymphocytes-monocytes (LMR). These ratios, the blood count data, CRP, and tHCY were log transformed (when appropriate) to normalize distributions. Outliers were identified and excluded using the extreme studentized deviate many-outlier procedure [18]. To account for differential weighting and the correlations within clustered data, we used SAS survey procedures (SURVEYMEANS, SURVEYFREQ, and SURVEYREG) with the DOMAIN option for analyzing subpopulations and used sampling weights according to NHANES guidelines [19]. We used linear regression models with counts and biomarkers as the dependent variables and reproductive events as the independent variables. We tabulated adjusted arithmetic or geometric count and biomarker means by reproductive events. Based on previously published data from NHANES [13–15], the following were considered potential confounders and adjusted for in the linear models: age (continuous), race (Mexican American, other Hispanic, non-Hispanic white, non-Hispanic black, other races), BMI (<18.5, 18.5–24.9, 25–29.9, 30–34.9, 35–39.9, ≥40), and current smoking (no, yes). Since laboratory methods varied over time, survey cycle (1999–2000, 2001–2, 2003–4, 2005–6, 2007–8, 2009–10, 2011–12) was also included. Because counts and biomarkers differed by menopausal status, the effects of reproductive events were examined separately for pre- and post menopausal women. Tests for trends for exposures in ordinal categories were calculated by creating an ordinal variable in which the median value or midpoint of each category was assigned to all participants in that group. For example, for the trend test for age at first live birth, the values 18, 22, 26, 31 and 37 were used to represent the categories <20, 20–24, 25–29, 30–34, and ≥35, respectively. These were the median ages within each category. This was done to minimize the effect of extreme values in the tails of the exposure distributions on the associations between continuous reproductive variables and biomarkers. P-values for categorical and trend variables were calculated with partial sum of squares F-tests.

## Results

Characteristics of the NHANES sample and their reproductive events are shown in Table 1. Women in the 2011–2012 cycle had lower average parity. Predictably, younger women were more likely to be nulliparous and have lower average parity, while older women were more likely to report later menarche, more children, have had a hysterectomy, or be menopausal. Non-Hispanic Blacks, Mexican-Americans and other Hispanics had: earlier menarche, earlier age at first birth, and more children. Accordingly, potential confounding by survey cycle, age, and race was considered when examining effects of reproductive events on the remaining variables in Table 1. Taller women reported later menarche and age at first birth. Heavier women had earlier menarche, more children, earlier age at first birth, and greater likelihood of having had a hysterectomy. Women who never smoked were more likely to be nulliparous. Current smokers reported more children and earlier ages at first and last birth. Former smokers were more likely to be menopausal and to have had a hysterectomy.

**Table 1:** Sample characteristics, NHANES 1999–2011.

	N (%)^2^	Age at menarche^1^	Nulliparous^1^	Parity^1^	Age at first live birth^1^	Age at last live birth^1^	Years since last live birth^1^	Hyster-ectomy^1^^,^^4^	Menopausal
		Mean (SE)	%	Mean (SE)	Mean (SE)	Mean (SE)	%	%	%
Data year									
1999–2000	2081 (13%)	12.8 (0.03)	20.0	2.2 (0.06)	22.3 (0.32)	28.3 (0.34)	21.1 (0.51)	26.3	42.4
2001–2002	2302 (15%)	12.7 (0.04)	19.6	2.2 (0.07)	22.9 (0.23)	28.9 (0.26)	20.9 (0.43)	24.9	41.7
2003–2004	2128 (14%)	12.7 (0.05)	23.1	2.0 (0.05)	22.7 (0.26)	28.3 (0.31)	22.1 (0.51)	22.5	42.4
2005–2006	2154 (14%)	12.7 (0.06)	21.0	2.1 (0.10)	23.0 (0.18)	28.8 (0.24)	21.5 (0.88)	21.6	41.8
2007–2008	2517 (15%)	12.7 (0.05)	18.0	2.1 (0.04)	not collected	not collected	not collected	21.6	43.2
2009–2010	2579 (14%)	12.8 (0.03)	18.9	2.1 (0.05)	not collected	not collected	not collected	21.0	43.8
2011–2012	2225 (15%)	12.8 (0.05)	22.0	1.9 (0.07)	not collected	not collected	not collected	22.9	46.7
p-value		0.60	0.13	0.007	0.34	0.34	0.34	0.16	0.48
Age									
20–24	1587 (9%)	12.5 (0.06)	64.3	0.6 (0.04)	19.0 (0.12)	20.8 (0.41)	1.9 (0.11)	0.1	0.2
25–34	2808 (17%)	12.5 (0.04)	33.0	1.4 (0.04)	22.1 (0.21)	25.9 (0.17)	4.4 (0.17)	1.6	0.9
35–44	2638 (19%)	12.7 (0.04)	14.9	2.0 (0.03)	23.6 (0.26)	29.0 (0.22)	10.6 (0.23)	13.9	7.0
45–54	2604 (21%)	12.8 (0.04)	12.0	2.1 (0.04)	23.5 (0.23)	29.1 (0.26)	20.2 (0.29)	25.3	41.8
55–64	2451 (15%)	12.7 (0.05)	12.2	2.4 (0.05)	22.1 (0.19)	28.8 (0.26)	30.6 (0.28)	36.6	96.6
65–74	2041 (10%)	12.9 (0.05)	8.9	3.1 (0.06)	22.1 (0.18)	29.9 (0.26)	39.4 (0.28)	48.0	100.0
≥75	1857 (9%)	13.1 (0.04)	10.6	3.1 (0.07)	23.3 (0.22)	32.0 (0.36)	48.2 (0.37)	47.8	100.0
p-trend		<0.0001	<0.0001	<0.0001	0.01	<0.0001	<0.0001	<0.0001	<0.0001
Race									
Mexican American	3038 (7%)	12.6 (0.04)	14.5	2.6 (0.05)	21.1 (0.15)	28.4 (0.21)	12.6 (0.48)	11.6	24.7
Other Hispanic	1213 (5%)	12.5 (0.08)	20.2	2.2 (0.08)	22.3 (0.38)	29.0 (0.46)	17.6 (0.96)	14.9	33.5
Non-Hispanic white	7698 (71%)	12.7 (0.02)	20.9	2.0 (0.03)	23.1 (0.18)	28.7 (0.18)	23.1 (0.37)	25.1	47.3
Non-Hispanic black	3172 (11%)	12.6 (0.04)	16.9	2.3 (0.04)	20.9 (0.14)	27.2 (0.20)	18.4 (0.43)	23.8	37.2
Other	865 (5%)	13.1 (0.08)	27.9	1.9 (0.06)	23.3 (0.35)	29.3 (0.46)	18.9 (0.87)	13.9	34.9
p-value		<0.0001	<0.0001	<0.0001	<0.0001	<0.0001	<0.0001	<0.0001	<0.0001
Height (cm)^3^									
<154.7	3146 (14%)	12.6 (0.05)	16.5	2.4 (0.04)	22.0 (0.17)	28.9 (0.19)	22.0 (0.19)	25.4	55.4
154.7–158.9	3145 (18%)	12.7 (0.04)	17.2	2.5 (0.04)	21.9 (0.19)	29.0 (0.26)	21.9 (0.24)	25.3	49.3
159–162.5	3058 (20%)	12.7 (0.05)	20.1	2.4 (0.04)	22.3 (0.17)	28.8 (0.19)	22.0 (0.20)	24.4	44.6
162.6–166.7	3205 (23%)	12.9 (0.04)	19.5	2.4 (0.04)	22.1 (0.20)	29.0 (0.23)	21.8 (0.24)	23.7	41.5
≥166.8	3246 (25%)	13.0 (0.05)	26.0	2.3 (0.04)	23.0 (0.23)	29.4 (0.26)	21.4 (0.27)	17.3	31.7
p-trend		<0.0001	0.49	0.02	0.0003	0.08	0.04	0.07	0.93
Weight (kg)^3^									
<58.2	2820 (18%)	13.1 (0.05)	28.7	2.2 (0.04)	22.7 (0.20)	29.1 (0.23)	22.0 (0.20)	17.8	39.2
58.2–66.6	3128 (21%)	13.0 (0.04)	20.7	2.4 (0.04)	22.5 (0.20)	28.9 (0.20)	22.0 (0.20)	21.3	41.8
66.7–75.7	3249 (20%)	12.7 (0.04)	18.4	2.5 (0.04)	22.4 (0.18)	29.2 (0.22)	21.8 (0.22)	23.0	45.3
75.8–89.2	3337 (21%)	12.6 (0.04)	16.6	2.5 (0.04)	21.7 (0.19)	29.0 (0.23)	21.9 (0.23)	25.3	47.1
≥89.3	3282 (20%)	12.4 (0.04)	18.8	2.5 (0.04)	21.7 (0.16)	29.0 (0.20)	22.0 (0.20)	25.9	41.4
p-trend		<0.0001	<0.0001	<0.0001	<0.0001	0.81	0.96	<0.0001	0.27
BMI (kg/m^2^)^3^									
<18.5	311 (2%)	13.4 (0.12)	34.9	2.1 (0.09)	22.2 (0.66)	28.1 (0.56)	22.7 (0.56)	17.1	33.4
18.5–24.9	4661 (34%)	13.1 (0.04)	26.0	2.2 (0.04)	23.0 (0.18)	29.2 (0.19)	21.7 (0.18)	17.7	36.0
25.0–29.9	4706 (29%)	12.8 (0.04)	17.0	2.4 (0.03)	22.1 (0.15)	29.0 (0.19)	21.9 (0.19)	24.5	48.1
30.0–34.9	3168 (18%)	12.5 (0.04)	14.8	2.5 (0.04)	21.7 (0.16)	28.9 (0.21)	21.9 (0.21)	26.5	48.0
35.0–39.9	1649 (10%)	12.4 (0.06)	15.5	2.6 (0.06)	21.8 (0.20)	28.9 (0.29)	21.9 (0.30)	27.6	43.6
≥40	1260 (7%)	12.3 (0.06)	23.6	2.4 (0.06)	21.9 (0.34)	28.8 (0.37)	22.0 (0.37)	25.2	44.2
p-trend		<0.0001	0.12	<0.0001	<0.0001	0.43	0.53	0.009	0.02
Smoking^3^									
Never	9874 (58%)	12.8 (0.03)	23.1	2.4 (0.03)	22.7 (0.12)	29.4 (0.15)	21.7 (0.15)	20.8	41.1
Former	3262 (22%)	12.7 (0.04)	15.6	2.3 (0.04)	22.4 (0.20)	28.9 (0.20)	22.1 (0.20)	28.8	56.6
Current	2836 (20%)	12.7 (0.05)	17.6	2.7 (0.04)	20.1 (0.18)	27.5 (0.21)	23.5 (0.21)	22.5	34.4
p-value		0.07	<0.0001	<0.0001	<0.0001	<0.0001	<0.0001	<0.0001	<0.0001

^1^Data were missing for age at menarche (n = 401), parity (n = 682), and hysterectomy (n = 373). Age at first, last, and years since last live birth was available for 6908, 6889, and 5605 parous women, respectively, in data years 1999–2006.

^2^Unweighted N and weighted percent.

^3^Adjusted for data year (1999–2000, 2001–2, 2003–4, 2005–6, 2007–8, 2009–10, 2011–12), age (continuous) and race (Mexican American, other Hispanic, non-Hispanic white, non-Hispanic black, other race).

^4^Hysterectomy in pre or postmenopausal women, with or without BSO.

Effects of reproductive evens on counts and biomarkers are shown in Tables 2 and 3. Age at menarche had no effects on counts among either pre- or postmenopausal women (Table 2). Compared to nulliparous premenopausal women, those who had given birth had lower monocytes, hemoglobin, and platelets; and, with increasing parity total WBC, neutrophils, and hemoglobin declined. Parous postmenopausal women had more lymphocytes, without any changes with increasing parity. An earlier age at first birth was associated with more platelets among premenopausal women and higher lymphocytes and monocytes among postmenopausal women. A later age at last livebirth was associated with lower WBC, neutrophils, monocytes, and platelets among premenopausal women but no changes in postmenopausal women. A longer interval since last birth was associated with more monocyte and platelets among preemnopausal women and lower hemoglobin among postmenopausal women. Premenopausal women who were currently using oral contraceptives (OCs) had higher lymphocytes, platelets, and hemoglobin and fewer monocytes. All counts were altered in women who were currently pregnant and reversed in women who were currently nursing. Hysterectomy was associated with higher hemoglobin and lower platelets among premenopausal women and more WBCs, lymphocytes, neutrophils, and platelets and lower hemoglobin among postmenopausal women. Postmenopausal women with longer term OC use had higher lymphocytes and those who were currently using estrogen-only HRT had more platelets. An early age at natural menopause was associated with more WBCs and neutrophils, lower lymphocytes, hemoglobin, and platelets.

**Table 2:** Adjusted WBC, differential and platelet count means for reproductive characteristics by menopausal status.

	N (%)^1^	WBC Mean^2^	Lymphocytes Mean^2^	Neutrophils Mean^2^	Monocytes Mean^2^	Hemoglobin Mean^2^	Platelets Mean^2^
**Menopausal status**							
Premenopausal	8401 (57%)	7.71	2.15	4.63	0.57	13.29	286.5
Postmenopausal	7585 (43%)	7.27	2.27	4.08	0.54	13.66	275.6
p-value		<0.0001	<0.0001	<0.0001	<0.0001	<0.0001	<0.0001
**Among premenopausal women**							
Age at menarche							
<11	788 (9%)	7.62	2.25	4.47	0.55	13.40	293.8
11	1092 (13%)	7.67	2.26	4.49	0.55	13.29	291.4
12–13	4207 (53%)	7.69	2.24	4.52	0.55	13.31	291.6
14–15	1673 (20%)	7.71	2.24	4.55	0.56	13.30	288.2
>15	456 (6%)	7.55	2.17	4.47	0.56	13.24	291.3
p-trend		0.86	0.08	0.64	0.11	0.11	0.25
Parity							
Nulliparous	1809 (28%)	7.64	2.25	4.47	0.57	13.48	298.9
Parous	6047 (72%)	7.64	2.24	4.49	0.55	13.29	289.1
p-value		0.93	0.55	0.76	0.0002	<0.0001	0.0003
Parity							
1	1662 (27%)	7.79	2.25	4.61	0.55	13.35	294.4
2	2119 (39%)	7.66	2.22	4.53	0.55	13.31	286.1
3	1413 (23%)	7.65	2.24	4.51	0.54	13.19	292.0
>3	853 (11%)	7.52	2.23	4.38	0.54	13.11	291.6
p-trend		0.02	0.80	0.02	0.18	<0.0001	0.67
Age at first live birth							
<20	1192 (29%)	7.74	2.27	4.54	0.56	13.33	306.1
20–24	1234 (36%)	7.85	2.30	4.63	0.56	13.36	301.6
25–29	628 (22%)	7.63	2.25	4.48	0.55	13.35	296.1
30–34	280 (10%)	7.70	2.27	4.52	0.55	13.45	292.2
≥35	65 (3%)	7.78	2.26	4.67	0.54	13.44	295.9
p-trend		0.63	0.74	0.82	0.22	0.28	0.02
Age at last live birth							
<25	1250 (32%)	7.91	2.28	4.68	0.57	13.41	304.3
25–29	973 (30%)	7.68	2.26	4.51	0.56	13.27	304.3
30–34	804 (26%)	7.69	2.30	4.49	0.55	13.33	301.3
≥35	371 (12%)	7.55	2.25	4.44	0.52	13.38	286.7
p-trend		0.03	0.94	0.04	<0.0001	0.68	0.02
Years since last live birth							
0–4	1411 (32%)	7.71	2.30	4.50	0.54	13.37	292.7
5–9	698 (21%)	7.77	2.27	4.58	0.56	13.37	296.8
10–15	645 (236%)	7.76	2.26	4.60	0.56	13.30	311.7
16+	644 (24%)	7.91	2.28	4.67	0.59	13.39	314.5
p-trend		0.31	0.73	0.27	0.002	0.99	0.0002
Oral contraceptive use							
Never	2607 (26%)	7.67	2.22	4.53	0.56	13.28	291.5
Former	4778 (60%)	7.68	2.25	4.51	0.55	13.35	290.8
Current	926 (14%)	7.73	2.32	4.54	0.53	13.43	300.0
p-value^3^		0.43	0.006	0.7	<0.0001	0.008	0.005
Pregnant at time of blood collection							
No	7256 (94%)	7.56	2.26	4.41	0.55	13.39	293.4
Yes	1145 (6%)	9.65	1.95	6.72	0.64	12.42	258.4
p-value		<0.0001	<0.0001	<0.0001	<0.0001	<0.0001	<0.0001
Nursing at time of blood collection							
No	8172 (98%)	7.70	2.23	4.55	0.55	13.32	290.8
Yes	229 (2%)	7.32	2.28	4.11	0.52	13.50	289.1
p-value		0.02	0.37	0.003	0.01	0.04	0.75
Hysterectomy							
No	7669 (95%)	7.67	2.24	4.52	0.55	13.29	292.2
Yes	384 (5%)	7.84	2.31	4.59	0.55	13.69	283.2
p-value		0.24	0.17	0.49	0.93	<0.0001	0.05
**Among postmenopausal women**							
Age at menarche							
<11	488 (7%)	7.21	2.15	4.08	0.56	13.46	267.0
11	894 (12%)	7.09	2.12	4.04	0.55	13.49	264.5
12–13	3631 (52%)	7.14	2.15	4.03	0.56	13.52	270.5
14–15	1745 (21%)	7.11	2.12	4.04	0.56	13.47	266.5
>15	611 (8%)	7.33	2.19	4.16	0.56	13.47	270.0
p-trend		0.35	0.71	0.37	0.95	0.63	0.89
Parity							
Nulliparous	727 (11%)	6.99	2.01	4.04	0.55	13.47	270.2
Parous	6721 (89%)	7.16	2.15	4.06	0.56	13.50	266.6
p-value		0.08	<0.0001	0.80	0.19	0.53	0.35
Parity							
1	891 (15%)	7.09	2.15	3.99	0.55	13.54	266.7
2	1836 (33%)	7.19	2.15	4.08	0.56	13.55	266.2
3	1563 (26%)	7.19	2.17	4.06	0.56	13.49	268.1
4	951 (12%)	7.07	2.09	4.01	0.56	13.50	266.8
>4	1480 (15%)	7.19	2.18	4.06	0.56	13.47	264.9
p-trend		0.86	0.98	0.83	0.26	0.10	0.78
Age at first live birth							
<20	1169 (31%)	7.27	2.24	4.07	0.58	13.62	275.3
20–24	1424 (42%)	7.05	2.16	3.95	0.57	13.70	272.8
25–29	625 (19%)	7.01	2.16	3.94	0.55	13.69	270.2
30–34	208 (6%)	7.10	2.13	4.02	0.57	13.71	273.4
≥35	83 (2%)	7.06	2.13	4.03	0.55	13.71	276.8
p-trend		0.10	0.02	0.45	0.003	0.23	0.60
Age at last live birth							
<25	672 (22%)	7.15	2.20	3.97	0.58	13.63	274.6
25–29	932 (30%)	7.08	2.20	3.96	0.57	13.63	271.0
30–34	932 (26%)	7.16	2.21	4.02	0.57	13.76	274.6
35–39	646 (16%)	7.17	2.17	4.06	0.57	13.64	271.1
≥40	309 (6%)	7.19	2.16	4.07	0.58	13.69	274.5
p-trend		0.57	0.38	0.20	0.76	0.28	0.89
Years since last live birth							
<28	870 (30%)	7.18	2.17	4.08	0.56	13.77	274.1
28–35	849 (24%)	7.06	2.17	3.96	0.57	13.75	273.1
36–43	878 (24%)	7.04	2.20	3.91	0.57	13.65	273.4
44+	894 (22%)	7.30	2.23	4.09	0.59	13.52	271.8
p-trend		0.72	0.39	0.85	0.13	0.004	0.76
Oral contraceptive use							
Never	3971 (45%)	7.37	2.19	4.23	0.56	13.61	266.1
≤5 years	2120 (31%)	7.34	2.19	4.19	0.57	13.71	270.7
>5–10 years	817 (13%)	7.38	2.23	4.22	0.56	13.64	267.4
>10 years	572 (10%)	7.42	2.28	4.16	0.57	13.54	272.8
p-value		0.64	0.04	0.58	0.77	0.19	0.23
Hormone replacement therapy							
Never used	4422 (54%)	7.39	2.19	4.24	0.56	13.64	266.8
Formerly used	1844 (29%)	7.39	2.23	4.20	0.57	13.67	272.0
Currently using estrogen only	705 (12%)	7.49	2.24	4.27	0.57	13.62	278.0
Currently using other hormones	251 (5%)	7.55	2.18	4.39	0.58	13.74	269.7
p-value, current estrogen only^4^		0.27	0.38	0.61	0.45	0.69	0.004
p-value, current other hormones^4^		0.29	0.68	0.16	0.19	0.18	0.86
Hysterectomy							
No	4242 (55%)	7.07	2.11	4.01	0.56	13.53	266.1
Yes	3318 (45%)	7.25	2.16	4.12	0.56	13.44	269.9
p-value		0.001	0.01	0.02	0.26	0.02	0.05
Age at natural menopause							
<46	1087 (23%)	7.47	2.14	4.34	0.56	13.42	262.8
46–49	918 (22%)	7.47	2.11	4.38	0.56	13.51	266.6
50–52	1330 (31%)	7.19	2.11	4.13	0.54	13.49	260.6
>52	1075 (25%)	7.14	2.10	4.08	0.55	13.53	266.0
p-trend		0.0004	0.30	0.0002	0.06	0.08	0.52

^1^ Unweighted N and weighted percent.

^2^ Adjusted for data year (1999–2000, 2001–2, 2003–4, 2005–6, 2007–8, 2009–10, 2011–12), age (continuous) and race (Mexican American, other Hispanic, non-Hispanic white, non-Hispanic black, other race), BMI (<18.5, 18.5–24.9, 25–29.9, 30–34.9, 35–39.9, ≥40), and current smoking (no, yes). Units are 1000 cells/uL for WBC, lymphocytes, neutrophils, monocytes, and platelets and g/dL for hemoglobin.

^3^ Current users compared to those who never or formerly used oral contraceptives.

^4^ Referent category is those who never or formerly used hormone replacement therapy.

**Table 3:** Adjusted inflammatory marker means for reproductive characteristics by menopausal status.

	Data available from 1999–2011							Data available from 1999–2010		Data available from 1999–2006	
	>N (%)^1^	PNR Mean^2^	PLR Mean^2^	PMR Mean^2^	NLR Mean^2^	NMR Mean^2^	LMR Mean^2^	N (%)^1^	CRP Mean^2^	N (%)^1^	tHCY Mean^2^
**Menopausal status**											
Premenopausal	8401 (57%)	62.2	131.0	539.3	2.11	8.67	4.12	7222 (58%)	0.26	4650 (58%)	7.61
Postmenopausal	7585 (43%)	66.6	119.6	546.2	1.80	8.20	4.57	6393 (42%)	0.30	3944 (42%)	8.02
p-value		0.002	<0.0001	0.50	<0.0001	0.003	<0.0001		0.01		0.001
**Among premenopausal women**											
Age at menarche											
<11	788 (9%)	63.8	126.7	573.1	1.99	8.98	4.52	667 (9%)	0.28	428 (9%)	6.80
11	1092 (13%)	62.9	124.9	567.5	1.99	9.03	4.54	931 (13%)	0.25	602 (13%)	6.56
12–13	4207 (53%)	62.4	125.9	564.0	2.02	9.03	4.48	3616 (53%)	0.26	2340 (54%)	6.55
14–15	1673 (20%)	61.3	124.7	551.9	2.03	9.00	4.43	1449 (20%)	0.26	895 (19%)	6.58
>15	456 (6%)	63.0	130.1	549.8	2.06	8.73	4.23	390 (6%)	0.28	241 (6%)	6.58
p-trend		0.24	0.52	0.04	0.13	0.35	0.004		0.77		0.52
Parity											
Nulliparous	1809 (28%)	65.0	129.2	565.1	1.99	8.69	4.37	1497 (27%)	0.25	973 (28%)	6.97
Parous	6047 (72%)	62.3	125.1	566.1	2.01	9.09	4.53	5272 (73%)	0.26	3382 (72%)	6.58
p-value		0.008	0.01	0.88	0.53	0.002	0.009		0.34		<0.0001
Parity											
1	1662 (27%)	61.9	126.9	572.0	2.05	9.24	4.51	1448 (27%)	0.31	980 (27%)	6.54
2	2119 (39%)	61.0	124.8	555.1	2.04	9.09	4.45	1825 (39%)	0.26	1152 (39%)	6.71
3	1413 (23%)	62.6	126.0	577.4	2.01	9.22	4.58	1248 (23%)	0.25	769 (23%)	6.70
>3	853 (11%)	64.5	126.8	574.8	1.96	8.91	4.53	751 (11%)	0.24	481 (12%)	6.57
p-trend		0.06	0.95	0.33	0.08	0.23	0.32		0.0001		0.55
Age at first live birth											
<20	1192 (29%)	65.4	131.0	583.2	2.00	8.91	4.45	1181 (29%)	0.27	1185 (29%)	6.83
20–24	1234 (36%)	63.2	127.2	573.4	2.01	9.07	4.51	1222 (36%)	0.27	1227 (36%)	6.53
25–29	628 (22%)	63.9	127.0	578.3	1.99	9.05	4.55	626 (23%)	0.26	626 (22%)	6.47
30–34	280 (10%)	62.3	124.0	562.5	1.99	9.03	4.54	279 (10%)	0.27	280 (10%)	6.43
≥35	65 (3%)	61.5	127.2	591.9	2.07	9.62	4.65	64 (3%)	0.23	64 (3%)	6.67
p-trend		0.18	0.12	0.60	0.98	0.25	0.30		0.50		0.05
Age at last live birth											
<25	1250 (32%)	63.0	129.2	564.5	2.05	8.96	4.37	1239 (32%)	0.29	1244 (32%)	6.78
25–29	973 (30%)	65.6	130.7	582.2	1.99	8.88	4.46	967 (30%)	0.27	971 (30%)	6.69
30–34	804 (26%)	65.1	126.9	592.5	1.95	9.10	4.67	797 (26%)	0.26	797 (26%)	6.41
≥35	371 (12%)	62.5	123.2	588.4	1.97	9.42	4.77	368 (12%)	0.22	369 (12%)	6.40
p-trend		0.87	0.08	0.06	0.09	0.07	0.0009		0.03		0.0005
Years since last live birth											
0–4	1411 (32%)	63.0	123.2	577.0	1.96	9.15	4.68	1401 (32%)	0.27	1408 (32%)	6.26
5–9	698 (21%)	62.8	126.7	569.0	2.02	9.06	4.49	692 (21%)	0.23	690 (21%)	6.64
10–15	645 (23%)	65.8	134.1	594.3	2.04	9.03	4.43	641 (23%)	0.27	644 (23%)	7.02
16+	644 (24%)	65.5	134.1	568.0	2.05	8.67	4.24	637 (24%)	0.31	639 (24%)	6.93
p-trend		0.14	0.004	0.90	0.22	0.05	0.003		0.09		0.0002
Oral contraceptive use											
Never	2607 (26%)	62.5	127.3	560.50	2.04	8.97	4.4	2203 (25%)	0.23	1393 (25%)	6.69
Former	4778 (60%)	62.3	125.1	561.60	2.01	9.01	4.49	4142 (60%)	0.25	2696 (60%)	6.61
Current	926 (14%)	64.1	125.5	613.90	1.96	9.58	4.89	796 (15%)	0.68	493 (14%)	6.66
p-value^3^		0.07	0.89	<0.0001	0.08	<0.0001	<0.0001		<0.0001		0.78
Pregnant at time of blood collection											
No	7256 (94%)	64.5	125.9	574.2	1.95	8.90	4.56	6135 (94%)	0.24	3673 (93%)	6.82
Yes	1145 (6%)	37.1	127.5	420.7	3.44	11.34	3.30	1087 (6%)	0.55	977 (7%)	4.64
p-value		<0.0001	0.38	<0.0001	<0.0001	<0.0001	<0.0001		<0.0001		<0.0001
Nursing at time of blood collection											
No	8172 (98%)	61.9	126	560.9	2.04	9.07	4.45	7016 (98%)	0.26	4505 (98%)	6.6
Yes	229 (2%)	68.1	122.7	597.7	1.80	8.77	4.87	206 (2%)	0.26	145 (2%)	6.78
p-value		0.005	0.39	0.05	0.004	0.34	0.01		0.88		0.31
Hysterectomy											
No	7669 (95%)	62.6	126.2	565.3	2.01	9.03	4.48	6559 (95%)	0.26	4137 (95%)	6.63
Yes	384 (5%)	59.6	118.6	546.2	1.99	9.17	4.61	326 (5%)	0.27	189 (5%)	6.82
p-value		0.04	0.01	0.21	0.69	0.54	0.27		0.57		0.28
**Among postmenopausal women**											
Age at menarche											
<11	488 (7%)	62.7	119.0	502.7	1.90	8.02	4.22	405 (7%)	0.31	223 (6%)	9.69
11	894 (12%)	62.9	119.9	508.5	1.91	8.09	4.24	760 (13%)	0.31	462 (13%)	9.16
12–13	3631 (52%)	64.5	121.0	513.3	1.88	7.96	4.24	3086 (52%)	0.32	1935 (53%)	9.17
14–15	1745 (21%)	63.2	120.6	508.8	1.91	8.05	4.22	1476 (21%)	0.33	874 (20%)	9.40
>15	611 (8%)	62.5	119.1	512.9	1.90	8.20	4.31	479 (7%)	0.34	313 (8%)	9.51
p-trend		0.50	0.88	0.79	0.61	0.40	0.69		0.20		0.21
Parity											
Nulliparous	727 (11%)	64.1	129.0	523.0	2.01	8.16	4.06	622 (11%)	0.31	434 (12%)	9.60
Parous	6721 (89%)	63.0	119.1	506.1	1.89	8.03	4.25	5678 (89%)	0.33	3500 (88%)	9.34
p-value		0.45	<0.0001	0.07	0.004	0.37	0.01		0.35		0.18
Parity											
1	891 (15%)	64.2	119.0	516.5	1.85	8.05	4.34	720 (15%)	0.32	441 (14%)	9.56
2	1836 (33%)	62.5	118.7	504.7	1.90	8.07	4.25	1499 (32%)	0.31	870 (30%)	9.43
3	1563 (26%)	63.3	118.4	507.1	1.87	8.02	4.28	1331 (25%)	0.34	787 (25%)	9.28
4	951 (12%)	63.8	122.4	508.8	1.92	7.98	4.16	805 (13%)	0.33	495 (13%)	9.35
>4	1480 (15%)	62.6	116.7	500.8	1.87	8.00	4.29	1323 (16%)	0.35	907 (18%)	9.35
p-trend		0.68	0.85	0.26	0.85	0.62	0.35		0.03		0.38
Age at first live birth											
<20	1169 (31%)	65.1	118.6	498.1	1.82	7.65	4.20	1143 (31%)	0.36	1156 (31%)	9.45
20–24	1424 (42%)	66.5	121.5	509.4	1.83	7.66	4.19	1396 (42%)	0.35	1411 (42%)	9.32
25–29	625 (19%)	66.0	120.4	520.4	1.82	7.89	4.32	619 (19%)	0.32	619 (19%)	9.18
30–34	208 (6%)	65.2	123.3	508.3	1.89	7.79	4.12	207 (6%)	0.29	207 (6%)	9.24
≥35	83 (2%)	65.9	124.9	538.6	1.90	8.17	4.31	79 (2%)	0.30	81 (2%)	9.28
p-trend		0.76	0.13	0.01	0.38	0.09	0.54		0.01		0.28
Age at last live birth											
<25	672 (22%)	66.7	120.2	505.6	1.80	7.58	4.21	661 (22%)	0.37	666 (22%)	9.54
25–29	932 (30%)	66.1	119.1	504.6	1.80	7.63	4.24	914 (30%)	0.34	918 (30%)	9.34
30–34	932 (26%)	65.6	119.3	511.2	1.82	7.79	4.28	916 (26%)	0.35	926 (26%)	9.16
35–39	646 (16%)	64.0	119.9	499.5	1.87	7.81	4.16	634 (16%)	0.34	641 (16%)	9.33
≥40	309 (6%)	64.9	122.1	504.7	1.88	7.77	4.13	301 (6%)	0.39	305 (6%)	9.63
p-trend		0.11	0.77	0.91	0.12	0.13	0.68		0.99		0.40
Years since last live birth											
<28	870 (30%)	64.3	120.9	514.8	1.88	8.00	4.26	855 (31%)	0.34	862 (30%)	9.41
28–35	849 (24%)	66.4	121.4	508.3	1.83	7.65	4.19	833 (24%)	0.35	839 (24%)	9.36
36–43	878 (24%)	67.4	119.8	510.0	1.78	7.57	4.26	863 (24%)	0.37	873 (24%)	9.01
44+	894 (22%)	64.1	117.6	488.7	1.83	7.62	4.16	875 (22%)	0.35	882 (22%)	9.63
p-trend		0.85	0.45	0.13	0.44	0.06	0.56		0.65		0.71
Oral contraceptive use											
Never	3971 (45%)	60.4	116.7	500.3	1.93	8.29	4.29	3438 (47%)	0.30	2302 (51%)	9.73
≤5 years	2120 (31%)	62.2	118.8	504.5	1.91	8.12	4.25	1750 (31%)	0.32	969 (29%)	9.5
>5–10 years	817 (13%)	61.1	115.8	507.6	1.90	8.31	4.38	671 (13%)	0.31	379 (12%)	9.45
>10 years	572 (10%)	63.5	115.8	508.3	1.82	8.01	4.39	449 (9%)	0.32	244 (8%)	9.60
p-value		0.14	0.41	0.37	0.04	0.41	0.09		0.51		0.47
Hormone replacement therapy											
Never used	4422 (54%)	60.5	117.3	502.8	1.94	8.31	4.29	3605 (52%)	0.29	2113 (48%)	9.81
Formerly used	1844 (29%)	62.4	117.7	505.1	1.89	8.09	4.29	1583 (29%)	0.30	909 (28%)	9.25
Currently using estrogen only	705 (12%)	62.6	119.4	513.9	1.91	8.21	4.31	653 (13%)	0.53	531 (17%)	8.68
Currently using other hormones	251 (5%)	59.3	119.2	492.2	2.01	8.29	4.13	242 (6%)	0.40	208 (8%)	8.73
p-value, current estrogen only^4^		0.20	0.31	0.21	0.77	0.90	0.83		<0.0001		<0.0001
p-value, current other hormones^4^		0.33	0.54	0.35	0.12	0.75	0.12		0.0007		<0.0001
Hysterectomy											
No	4242 (55%)	63.8	121.1	509.5	1.90	7.98	4.21	3544 (55%)	0.29	2143 (53%)	9.41
Yes	3318 (45%)	62.7	119.6	509.8	1.91	8.13	4.26	2829 (45%)	0.38	1791 (47%)	9.32
p-value		0.12	0.23	0.94	0.69	0.15	0.13		<0.0001		0.41
Age at natural menopause											
<46	1087 (23%)	58.0	117.7	495.3	2.03	8.53	4.21	923 (23%)	0.36	532 (23%)	9.86
46–49	918 (22%)	58.4	121.3	501.6	2.08	8.59	4.13	771 (22%)	0.37	473 (24%)	9.78
50–52	1330 (31%)	60.6	118.4	510.4	1.95	8.42	4.31	1094 (30%)	0.30	678 (31%)	9.50
>52	1075 (25%)	62.8	121.9	516.2	1.94	8.22	4.23	910 (25%)	0.34	546 (23%)	9.13
p-trend		<0.0001	0.13	0.01	0.04	0.06	0.49		0.26		0.0002

^1^ Unweighted N and weighted percent.

^2^ Adjusted for data year (1999–2000, 2001–2, 2003–4, 2005–6, 2007–8, 2009–10, 2011–12), age (continuous) and race (Mexican American, other Hispanic, non-Hispanic white, non-Hispanic black, other race), BMI (<18.5, 18.5–24.9, 25–29.9, 30–34.9, 35–39.9, ≥40), and current smoking (no, yes). Units are mg/dL for CRP and umol/L for tHCY.

^3^ Current users compared to those who never or formerly used oral contraceptives.

^4^ Referent category is those who never or formerly used hormone replacement therapy.

Premenopausal women with an earlier age at menarche had higher PMR and LMR (Table 3). Compared to nulliparous premenopausal women, those who had given birth had lower PNR, PLR, and tHCY and higher NMR, and LMR; and, with increasing parity, CRP decreased. Parous postmenopasual women had lower PLR and NLR and greater LMR; and, with increasing parity, CRP increased. An earlier age at first birth was associated with higher tHCY among premenopausal women and lower PMR and higher CRP among postmenopausal women. Among premenopausal women, a later age at last livebirth was associated with higher LMR and lower CRP and tHCY; and PLR and tHCY increased and LMR decreased with more years since last birth. Among postmenopausal women no changes in biomarker were seen with either of these events. Premenopausal women who were currently using OCs had higher PMR, NMR, LMR, and CRP. Similar to the story for counts, virtually all biomarkers were altered in women who were currently pregnant and reversed in women who were currently nursing. Hysterectomy was associated with lower PNR and PLR among premenopausal women and with higher CRP among postmenopausal women. Longer term past use of OCs was associated with lower NLR among postmenopausal women while current use of estrogen only HRT was associated with higher CRP and lower tHCY. Early age at natural menopause was associated with lower PNR and PMR and higher NLR and tHCY.

The results presented in Tables 2 and 3 are summarized in Table 4. In this table, the reproductive events are shown in the second column for pre- and postmenopausal women separately. An upward arrow indicates the event raised the count or biomarker and a downward arrow indicates the event lowered the count or biomarker. Events which significantly affected the counts or biomarkers are indicated by a bold arrow for an association with p value <0.0003 and a non-bold arrow for p values ≥0.0003 and ≤0.05. A p-value of 0.0003, represents a Bonferroni-corrected p based on 12 inflammatory biomarkers from the blood count data and the 12 reproductive variables.

**Table 4:** Effect of specified reproductive event on counts and biomarkers in pre- and postmenopausal women.

Menopausal status	Event	WBC	L	N	M	HGB	P	PNR	PLR	PMR	NLR	NMR	LMR	CRP	tHCY
Premenopausal	Decreasing age at menarche									↑			↑		
	Nulliparity				**↑**	**↑**	↑	↑	↑			↓	↓		**↑**
	Increasing parity	↓		↓		**↓**								**↓**	
	Early age at first live birth						↑								↑
	Late age at last live birth	↓		↓	**↓**		↓						↑	↓	↓
	Shorter time since last pregnancy				↓		**↓**		↓				↑		**↓**
	Current OC use		↑		**↓**	↑	↑			**↑**		**↑**	**↑**	**↑**	
	Pregnant	**↑**	**↓**	**↑**	**↑**	**↓**	**↓**	**↓**		**↓**	**↑**	**↑**	**↓**	**↑**	**↓**
	Nursing	↓		↓	↓	↑		↑			↓		↑		
	Hysterectomy					**↑**		↓	↓						
Postmenopausal	Decreasing age at menarche														
	Nulliparity		**↓**						**↑**		↑		↓		
	Increasing parity													↑	
	Early age at first live birth		↑		↑					↓				↑	
	Late age at last live birth														
	Shorter time since last pregnancy					↑									
	Longer duration of OC use		↑								↓				
	Current estrogen only HRT use													**↑**	**↓**
	Hysterectomy	↑	↑	↑		↓								**↑**	
	Early age at natural menopause	↑		**↑**				**↓**		↓	↑				**↑**

↑ and ↓ indicate count or maker increased or decreased, respectively, with the event in column 1.

Abbreviations: WBC: white blood cell, L: lymphocytes, N: neutrophils, M: monocytes, HGB: hemoglobin, P: platelets, PNR: platelet:neutrophil ratio, PLR: platelet:lymphocyte ratio, PMR: platelet:monocyte ratio, NLR: neutrophil:lymphocyte ratio, NMR: neutrophil:monocyte ratio, LMR: lymphocyte:monocyte ratio, CRP: C-reactive protein, HCY: homocysteine, OC: oral contraceptive, HRT: hormone replacement therapy.

**↑** or **↓** p-values <0.0003

↑ or ↓ p-values ≥0.0003 and <0.05

Finally, Table 5 shows correlations of counts and ratios with CRP and tHCY. For counts, the strongest positive correlations with CRP were with neutrophils and platelets. Surprisingly, CRP was inversely correlated with PNR suggesting that more platelets relative to neutrophils signals lower inflammation. Positive correlations were also seen between CRP and NLR, and NMR. For both counts and biomarkers derived from counts, correlations with CRP were present and in the same direction in both pre- and postmenopausal women. In all women, the strongest positive correlations with tHCY were inverse correlations with neutrophils, NMR, and NLR and a positive correlation with PNR. In all of these instances, the correlations were stronger in premenopausal women with opposite or null correlations in postmenopausal women.

**Table 5:** Pearson correlations between differential and platelet counts and their ratios and CRP and tHCY.

		All women		Premenopausal		Postmenopausal	
		CRP	tHCY	CRP	tHCY	CRP	tHCY
Lymphocytes	r	0.10	-0.01	0.10	0.11	0.12	-0.09
	p	<.0001	0.29	<.0001	<.0001	<.0001	<.0001
Neutrophils	r	0.29	-0.21	0.32	-0.29	0.29	0.09
	p	<.0001	<.0001	<.0001	<.0001	<.0001	<.0001
Monocytes	r	0.14	-0.03	0.13	-0.14	0.15	0.10
	p	<.0001	0.01	<.0001	<.0001	<.0001	<.0001
Hemoglobin	r	-0.07	0.13	-0.11	0.21	-0.05	-0.17
	p	<.0001	<.0001	<.0001	<.0001	<.0001	<.0001
Platelets	r	0.18	0.01	0.19	0.16	0.20	-0.04
	p	<.0001	0.43	<.0001	<.0001	<.0001	0.01
PNR	r	-0.17	0.19	-0.19	0.34	-0.15	-0.11
	p	<.0001	<.0001	<.0001	<.0001	<.0001	<.0001
PLR	r	0.03	0.01	0.04	0.02	0.02	0.05
	p	0.0004	0.20	0.0002	0.17	0.16	0.002
PMR	r	-0.002	0.03	0.01	0.22	0.00	-0.12
	p	0.80	0.02	0.62	<.0001	0.91	<.0001
NLR	r	0.18	-0.17	0.22	-0.32	0.14	0.14
	p	<.0001	<.0001	<.0001	<.0001	<.0001	<.0001
NMR	r	0.18	-0.19	0.23	-0.19	0.15	0.01
	p	<.0001	<.0001	<.0001	<.0001	<.0001	0.76
LMR	r	-0.03	0.01	-0.03	0.21	-0.02	-0.16
	p	0.0004	0.23	0.004	<.0001	0.21	<.0001

## Discussion

This analysis of cross-sectional data from NHANES reveals that reproductive events have both short and long-term effects on blood counts, ratios of those counts, and the serum biomarkers, CRP and tHCY. This study was prompted by observations that reproductive events have major effects on chronic disease risk that may not be entirely explained by traditional interpretations involving estrogen levels. Our discussion is organized around the following topics: effects of reproductive events on counts and biomarkers in “real time”; residual effects of reproductive event on counts and biomarkers and possible relevance to chronic disease risk; and potential limitations of the study. We will conclude with an overview and suggestions for future studies.

### Real time effects of reproductive events

NHANES participants included women who were currently pregnant, breastfeeding or using hormones allowing the effects of these events on counts and biomarkers to be studied in real time. Pregnancy affected virtually all counts and biomarker, confirming that pregnancy is an intensely “pro-inflammatory” state [20]. Most of the changes observed during pregnancy were reversed in mothers who were nursing. Premenopausal women who were using OCs had several changes in counts and biomarkers including elevated CRP. The link with CRP has been previously reported and may explain greater risk of thromboembolism in OC users[21]. Postmenopausal women using estrogen-only HRT also had elevated CRP which also has been reported previously [22].

### Residual effects

Reproductive events also left residual effects on counts and inflammatory markers which may be relevant to how menarche, menopause, and childbearing affect chronic disease risk.

#### Menarche

Early menarche is associated with increased risk of mortality from all-causes and CVD [23, 24], breast cancer [25], and type II diabetes [26]. Obesity is an important confounder for these associations since early menarche predicts obesity in later life [27]. Correction for BMI attenuates risk for diabetes associated with early menarche [28, 29] and may partly explain associations with cardiovascular disease [30] and breast cancer [25]. Adjusting for BMI and age, early menarche has not been associated with higher estrogen levels in later life in most studies [31–34]. In this study, an earlier age at menarche was associated with higher PMR and LMR, but only in premenopausal women.

#### Menopause

Occurrence of menopause affected nearly all counts and inflammatory biomarkers. Except for lymphocytes and hemoglobin, counts were lower in postmenopausal women and most markers were also lower, except PNR and LMR. Women with an earlier age at natural menopause had higher WBC and neutrophil counts and tHCY. This profile is known to increase the risk for CVD and mortality from all causes [1, 4, 35] and could help explain their association with early menopause [36, 37], especially since there is little evidence that age at or time since menopause have any effects on future estrogen levels[31, 32, 34, 38, 39]. As opposed to CVD risk, early menopause decreases the risk for breast and endometrial cancer [40].

#### Childbearing

A review of studies worldwide [41] found no consistent agreement on how childbearing affects CVD risk. However, a study of U.S. cohorts concluded that risk for CVD and coronary artery disease increased after 5 pregnancies after adjusting for age, educational level, and weight [42]. Another large cohort study suggested the association may be J-shaped with higher risk for nulliparous women, a nadir in risk for women with 2 children and increasing risk with more pregnancies, especially after 5 [43]. Potentially relevant to these observations are higher tHCY levels for nulliparous premenopausal women and higher NLR and PLR for nulliparous postmenopausal women. In addition, with more children CRP levels increased in postmenopausal women but decreased among premenopausal women. Epidemiologic data are more consistent for parity and cancer occurrence or mortality. With notable exceptions for liver, cervix, and myeloma, nulliparous women are at greater risk for death from most types of cancers, especially breast, endometrium, and ovary; and risk further decreases with increasing parity [44–46]. Clearly relevant is the observation that parous women have lower estradiol and estrone levels than nulliparous women and, among postmenopausal women, the levels may further decline with increasing number of children [33, 34, 47]. However, possibly relevant, we found higher PLR and lower LMR in pre- and postmenopausal women who were nulliparous. Higher PLR and lower LMR predict worse survival after breast cancer [8, 48, 49].

Early age at first birth is associated with decreased risk for breast cancer—consistent with observations that early age at first birth is associated with lower estrone and bioavailable estradiol levels among postmenopausal women [34]. First birth delayed after age 35 is less protective (or may actually increase risk) [50–52]. Lambe et al. studied women with 2 or more pregnancies and found that breast cancer risk increased with both advancing age at first birth and advancing age at last birth[53]. Elevated risk for breast cancer was more apparent in women with an older age at and shorter interval since last birth—compatible with the theory there is a transient increase in risk after any pregnancy which decreases and then becomes protective with more time since pregnancy [54]. In our study, an earlier age at first birth was associated with lower PMR and higher monocytes and CRP among postmenopausal women. Among premenopausal women, later age at and shorter interval since last birth was most clearly associated with lower monocytes and higher LMR. Turning to CVD risk, research from the Nurses’ Health Study, mostly involving women less than 60, found no effect of an early age at first livebirth [55]. However, in an international study, first birth during adolescence was associated with greater risk for diabetes, lung disease, and hypertension [56]. In our study, an early age at first birth was associated with higher tHCY in premenopausal women and with more monocytes in postmenopausal women. Notably, a Framingham study found that, among leukocyte subsets, elevated monocytes were the best predictor of future CVD risk [57].

### Study limitations

Misclassification, confounding, and false discovery are issues that should be considered in interpreting the findings of this study. Although any self-reported data are subject to misclassification, reproductive events are memorable landmarks for women. In surveys repeated over time, 75 to 95% of women correctly recalled within a year their previously reported ages at menarche and natural or surgical menopause [58]. Based on prior observations from NHANES, important confounders in examining the effects of reproductive events on blood counts or inflammatory biomarkers include age, race, BMI, and current smoking and were adjusted for in our analyses. The large size and number of variables in the NHANES sample used here provides considerable power to detect even small effects of reproductive events on counts and inflammatory biomarkers. However, this also increases the likelihood of false positives; hence we highlighted those associations with a Bonferroni-corrected p value of 0.0003 in Table 4.

#### Summary and future research

Epidemiologic evidence reviewed here reveals that reproductive events have substantial impact on risks for chronic diseases, especially cardiovascular diseases and cancers of the reproductive organs. It is intriguing that reproductive events often track differently in their impact on CVD and cancer risk. Our analysis of NHANES data reveals that reproductive events affect blood counts and inflammatory biomarkers in real time as well as leaving residual effects. The fact that these changes are seen with simple morphologic descriptors of white blood cell types suggests that further characterization of WBCs (e.g. T cells, B cells, NK cells, etc.) could be informative. Focusing on the effects of reproductive events on specific white cell types and the ratios derived from them in Table 4, it can be seen that 16 of the 20 bold arrows involve either monocytes or platelets or ratios that include monocytes or platelets highlighting the potential importance of these two compartments. Relevant to this are studies of platelets as immune cells and platelet-monocyte aggregates in coronary artery disease and cancer[59–61]. Clarifying the role of oxidative stress and lipoprotein receptors on a variety of cells may help further bridge the connections between inflammation, immunity, atherogeneisis, and tumorigneisis[62]. Further characterization of WBC subclasses and studies of the effects of oxidative stress on platelets, monocytes, and the tissues they interact with may reveal exactly how reproductive events mediate risk for a variety of chronic diseases expanding on traditional interpretations involving hormone levels.
